# International commentary: Child injuries on the farm: A brief commentary from Canada

**DOI:** 10.3389/fpubh.2022.1050621

**Published:** 2022-11-28

**Authors:** William Pickett, Kathy L. Belton, Andrea Lear, Robin Anderson, Donald C. Voaklander

**Affiliations:** ^1^Department of Health Sciences, Brock University, St. Catharines, ON, Canada; ^2^Department of Public Health Sciences, Queen's University, Kingston, ON, Canada; ^3^School of Public Health, Injury Prevention Centre, University of Alberta, Edmonton, AB, Canada; ^4^Canadian Agricultural Safety Association, Winnipeg, MB, Canada

**Keywords:** agriculture, Canada, child health, epidemiology, farming, injury, occupational health and safety, pediatrics

## Introduction

It has long been established that children on farms are especially vulnerable to major injury. This is true in much of the world (1, 2), including our own country of Canada (3, 4). It is also true of children who grow up as members of farm families who reside on farm and ranch (i.e., large farm) properties (5), as well as children who frequent farms as occasional workers (6) or as visitors to the farm and its related worksites (7). In Canada, there is potential for children to be exposed to a diversity of mechanical, structural, chemical and other physical hazards associated with agricultural production activities (8, 9). Such hazards present risks for major trauma, injury, and disability (1–9). Patterns of injury evolve as children grow, develop and begin to take on essential work roles as part of the farm operation.

In this commentary we reflect on the current state of knowledge about the injury problem on Canadian farms. Our hope is to briefly summarize the current state of epidemiological evidence surrounding the child farm injury problem in Canada using evidence from our ongoing, national surveillance program (9). Building on this foundation, we will present evidence and opinion derived from international research about what works to prevent farm injuries among different groups of children. We will highlight the existence of policies and programs that are available within the Canadian agricultural sector and summarize barriers to the implementation of known effective strategies from the perspectives of health and safety professionals (e.g., Agricultural Safety Program Coordinators, Health and Safety Advisors, Farm Safety Consultants and Educators). who work with the farming community on an ongoing basis.

## Fatal farm injuries to children: What we know

While agriculture is evolving in Canada with more corporate farms emerging in recent years, most farms continue to operate as family farms (“sole propriertorships” and “partnerships”) with a heavy emphasis on the raising of grain and large animal products (10). In terms of injury, nationally representative data on the most serious types of farm injury are available from the Canadian Agricultural Injury Reporting program (11), our national system for the surveillance of injuries related to agriculture. Available data include reports from 1990 to present on fatalities related to agricultural work and settings, including those involving children (Table 1). Trends and patterns within these fatality records are informative. Overall, while annual counts and number of children at risk have declined as the agricultural sector has changed, on a per capita basis the risks to farm children have remained fairly constant over the past three decades (11). The majority of child fatalities are experienced by boys who are residents of farms. Most involve machinery, primarily tractors due to runovers and rollovers, with emergent risks associated with ATV operation (11). Preschool-aged children (11, 12) and young workers (6) are particularly vulnerable, especially when assigned to situations and tasks that they are developmentally incapable of handling (13–15), and with inadequate adult supervision (16). This situation has persisted for generations on Canadian farms, and while much has changed due to advances in work and technology (8), much remains the same.

**Table 1:** Fatal farm injuries to children 0 to 14 years in Canada from 1990 to 2022 (*n* = 318 fatalities)^*^.

**Number of resident children**	
**Annual rate of fatal farm injuries**	4.7 per 100,000
**Fatalities to boys**	78%
**Fatalities to preschool-aged children (< ** **age 6)**	53%
**Fatalities to children of farm operators**	70%
**Machinery involved**	65%
**Work involved**	71%
**Victim working**	17%
**Top 3 machines involved**	Tractors (45%)
	ATVs (13%); Other Motor vehicles (11%)
**Top 3 non-machinery causes**	Drowning (37%)
	Animal trauma (14%)
	Struck by objects (13%)

* Source: Canadian Agricultural Injury Reporting (9).

## Child farm injury in Canada: Some perspective

Tradition, culture and economic demands within farm populations are important factors in understanding why children continue to be exposed to farm hazards and associated risks for injury. Farming in North America in many ways is a unique occupation. Farm operators balance the need to keep themselves, their families and their workers safe from occupational hazards while simultaneously trying to pass on important values within their families, and while trying to maximize productivity and associated financial returns in the face of economic uncertainty (17–19). Consequences of this situation are well understood. Positively, children are intentionally exposed to situations that put them at risk to promote the values of hard work and autonomy, to advance their growth and development, and to foster strong ties between generations of families (18). All of these are good. More negatively, these values and practices can also lead to an acceptance of risk for children and young workers that is problematic, because it leads to the fatalities described above (11, 12, 16). Solutions to the injury problem on North American farms are unlikely to be effective if imposed from government or industry without the direct involvement of farmers. Farmers have historically resisted occupational health and safety regulations as an affront to their autonomy (19–21). What is valued culturally by most of the Canadian farm community often is in conflict with best practices for prevention from the public health, clinical and health and safety communities. This is an important challenge for health and safety efforts.

## Prevention strategies: The evidence base

In this commentary, we have drawn upon available systematic reviews (21, 22) to describe what is known about the effectiveness of various strategies to prevent injury on farms. There are few randomized trials or other sophisticated evaluations of different strategies to prevent child injury on farms available in the scientific literature. The most widely accepted strategies are educational in nature, and these tend to be effective in making people aware of hazards, but with limited demonstration that they ultimately change rates of major trauma. Engineered solutions are also common and effective, but not universally adopted due to very real considerations of cost and the challenge of regulating occupational worksites that are autonomous and geographically dispersed. Policy solutions (referred to as “regulations” or “enforcement” in the injury prevention literature) too are available *via* occupational health and safety legislation applying to child labor practices (16) and highly effective when enforced (16). Yet, such legislation sometimes does not apply to resident farm children, nor does it cover situations where children are brought in to the workplace when adults are working, a circumstance common to many child fatalities (11, 12, 16).

## Barriers to implementation of effective prevention strategies: An honest reflection

The prevention of traumatic injury on farms and ranches remains a major challenge. In the development of this commentary, front-line agricultural safety and health practitioners associated with the Canadian Agricultural Safety Association were consulted to seek their expertise on barriers to the implementation of safety strategies that are known to be effective in other industries. A summary follows.

Agricultural work contexts are thought to be unique. The motivations and passion behind these agricultural businesses are steeped in tradition (19, 20) and are often not motivated solely for financial gain—for the most part, people are farming and ranching because of the lifestyle and passion for the occupation. Many farms and ranches are also the location of family homes, meaning there is a mix of “workplaces” and “play places”.

“*It's a deeper question than just covering regulations – it's a mind-set. Separating work from family on the farm becomes blurred when the workplace is also a living space and a playground*.” Canadian Agricultural Safety and Health Practitioner (Anonymous), 2022.

Engineered solutions, including the maintaining of equipment and structures, and the creation and maintenance of safe play areas (23) are considered vital, but can be expensive. Education solutions, including teaching producers about age-appropriate work (24), and children and youth from a young age about health and safety (25) are similarly held in high regard. Policy solutions like daycare (20, 21), farm safety audits (8), regulatory enforcement of child labor practices (5), and (although not studied in detail) financial incentives to improve farm safety (26) may be most effective, but can be divisive. In practice, there is considerable support for self/farm family-driven solutions (e.g., self-directed safety audits; grassroots childcare initiatives). There is considerably less support for imposed solutions like regulatory enforcement.

“*In general, the introduction of legislation is always overwhelming for farm businesses. Farms have a tremendous amount of regulatory and administrative burdens, which are identified as one of the primary stressors contributing to mental health challenges in agriculture*.” Canadian Agricultural Safety and Health Practitioner (Anonymous), 2022.

## Final thoughts

Traumatic farm injuries to children remain an important public health problem in Canada. Producers, communities, and agriculture-based organizations in our country, including safety and health organizations, do embrace keeping children and youth safe. Farm families in Canada are acutely aware of hazards associated with farm life and make adjudicated decisions to balance the risks and benefits of intentional farm and farm work exposures (18). Moreover, there is agreement that it is important that the sustainability of family farms and ranches is supported, and this includes protecting farm families.

Solutions are complex and are ultimately influenced by tradition. Ultimately, the prevention of injuries to children on Canadian farms will necessarily involve a culture change, including a shift in the narrative on how children and youth live and play on farms and ranches, with the support of evidence-based education, engineering and policy solutions. This remains an ongoing challenge for all concerned.

## Author contributions

WP prepared the initial draft of this manuscript. AL and RA surveyed agricultural safety professionals from across Canada in order to summarize their insights about the prevention of injuries to children on farms. KB, AL, RA, and DV reviewed and edited the manuscript for important intellectual content. DV and KB oversaw the analysis that underlies this commentary. All authors contributed to the article and approved the submitted version.

## Conflict of interest

The authors declare that the research was conducted in the absence of any commercial or financial relationships that could be construed as a potential conflict of interest.

## Publisher's note

All claims expressed in this article are solely those of the authors and do not necessarily represent those of their affiliated organizations, or those of the publisher, the editors and the reviewers. Any product that may be evaluated in this article, or claim that may be made by its manufacturer, is not guaranteed or endorsed by the publisher.
